# Reply to the letter: autonomy and refusal of dialysis: ethical and legal reflections on the Brazilian Society of Nephrology Position Statement

**DOI:** 10.1590/21758239-JBN-2025-0326rpen

**Published:** 2026-07-24

**Authors:** Dirceu Reis da Silva, Fernanda Salomão Gorayeb Polacchini, Ana Flávia Moura, Cibele Isaac Saad Rodrigues, Maurício Younes-Ibrahim, Eduardo Rocha, Marina Aline Occhiena de Oliveira Neiva, Pedro Túlio Rocha, Patrícia Ferreira Abreu, José A Moura-Neto

**Affiliations:** 1 Sociedade Brasileira de Nefrologia, São Paulo, SP, Brazil.; 2 Hospital de Clínicas de Porto Alegre, Porto Alegre, RS, Brazil.; 3 Instituto de Doenças Renais, Porto Alegre, RS, Brazil.; 4 Hospital de Base de São José do Rio Preto, Faculdade de Medicina de São José do Rio Preto, São José do Rio Preto, SP, Brazil.; 5 Escola Bahiana de Medicina e Saúde Pública, Salvador, BA, Brazil.; 6 Pontifícia Universidade Católica, Faculdade de Ciências Médicas e da Saúde, Sorocaba, SP, Brazil.; 7 Hospital Santa Lucinda, Sorocaba, SP, Brazil.; 8 Universidade do Estado do Rio de Janeiro, Rio de Janeiro, RJ, Brazil.; 9 Pontifícia Universidade Católica do Rio de Janeiro, Rio de Janeiro, RJ, Brazil.; 10 Renalle Consultoria e Serviços Médicos, Petrópolis, RJ, Brazil.; 11 Universidade Federal do Rio de Janeiro, Rio de Janeiro, RJ, Brazil.; 12 Hospital Santa Casa de Misericórdia de Goiânia, Goiânia, GO, Brazil.; 13 Hospital do Rim, Goiânia, GO, Brazil.; 14 Hospital São Lucas Copacabana, Rio de Janeiro, RJ, Brazil.; 15 Universidade Federal de São Paulo, São Paulo, SP, Brazil.

Dear Editor,

The publication of the Brazilian Society of Nephrology’s Position Statement on the refusal and discontinuation of dialysis^1^ fills a gap in the national nephrology literature, providing a technical and ethical framework for situations in which treatment no longer seems proportionate or beneficial to the patient. The document brings greater certainty for processes marked by suffering, anguish, uncertainty, and potential conflicts, in which technical responsibility, beneficence, non-maleficence, justice, and respect for the dignity and autonomy of the person with advanced kidney disease are intertwined^2^.

Dialysis cannot be an absolute imperative but an intervention whose proportionality must be reassessed in light of the patient’s clinical condition, quality of life, and beliefs and values. Physicians should not limit themselves to the technical dimension but plan care that recognizes the patient as a bioethical being and not merely a recipient of procedures.

The analysis by Santos et al.^3^ rightly highlights the autonomous and legitimate nature of decisions regarding medical interventions when made by the person who matters most: the patient themselves. By warning that the compulsive pursuit of consensus can obscure and even harm this prerogative, they reinforce a robust conception of autonomy, which is not reduced to signing a document but involves informed self-determination, consistent with beliefs, values, and life projects. In line with international guidelines and a recently enacted national law^4^, the right to refuse or discontinue therapies that do not align with care objectives is emphasized^5^.

However, patient autonomy can create tension with the team’s ethical and technical shared responsibility. The nephrologist is responsible for the adequacy of the indication and for assessing the pertinence or disproportionality of the interventions. When respecting the patient’s decision not to initiate or to discontinue dialysis, they need to ensure that decision-making capacity, understanding, and freedom of choice are present, and also that adequate symptomatic and palliative support will be provided.

Informed refusal is undoubtedly respectable, while refusal based on misinformation, ignorance, desperation, or subtle coercion requires (bio)ethical intervention from the professional and the team. Thus, it is necessary to ensure that the decision-making process is shared, with honest communication, active listening, and transparent documentation of the patient’s preferences. Clinical caution is desirable to avoid incompetence, recklessness, or negligence if there is no consistent offer of care alternatives. The team must respect legitimate refusal, as well as consider that uremia compromises autonomy, contributing to the patient’s emotional and cognitive vulnerability, which is not always evident.

In conclusion, analyses from diverse perspectives are welcome and contribute to improving particularly difficult decision-making processes, in which the team, the patient, and their family need to weigh therapeutic proportionality, suffering, prognosis, personal values, and care objectives. Thus, while the SBN’s Position Statement values patient autonomy and proposes consensus as a procedural ideal, it also offers a framework that protects the shared responsibility of the team, allowing for the prudent and morally courageous recognition of when interrupting dialysis may, in fact, mean providing better care.

## Data Availability

No new data were generated or analyzed in this study.
