# Supplementary material for: Reply to the letter: autonomy and refusal of dialysis: ethical and legal reflections on the Brazilian Society of Nephrology Position Statement
Source: J Bras Nefrol. 2026 Jul 24;48(3):e20250326rp. doi: 10.1590/21758239-JBN-2025-0326rpen (PMC13427307; doi:10.1590/21758239-JBN-2025-0326rpen)
Supplement: Translation

# Translation: Resposta à carta: autonomia e recusa da diálise: reflexões éticas e jurídicas a partir do posicionamento da Sociedade Brasileira de Nefrologia

**DOI:** 10.1590/21758239-JBN-2025-0326rppt

**Authors:** Dirceu Reis da Silva, Fernanda Salomão Gorayeb Polacchini, Ana Flávia Moura, Cibele Isaac Saad Rodrigues, Maurício Younes-Ibrahim, Eduardo Rocha, Marina Aline Occhiena de Oliveira Neiva, Pedro Túlio Rocha, Patrícia Ferreira Abreu, José A Moura-Neto

**Affiliations:** 1 Sociedade Brasileira de Nefrologia, São Paulo, SP, Brasil.; 2 Hospital de Clínicas de Porto Alegre, Porto Alegre, RS, Brasil.; 3 Instituto de Doenças Renais, Porto Alegre, RS, Brasil.; 4 Hospital de Base de São José do Rio Preto, Faculdade de Medicina de São José do Rio Preto, São José do Rio Preto, SP, Brasil.; 5 Escola Bahiana de Medicina e Saúde Pública, Salvador, BA, Brasil.; 6 Pontifícia Universidade Católica, Faculdade de Ciências Médicas e da Saúde, Sorocaba, SP, Brasil.; 7 Hospital Santa Lucinda, Sorocaba, SP, Brasil.; 8 Universidade do Estado do Rio de Janeiro, Rio de Janeiro, RJ, Brasil.; 9 Pontifícia Universidade Católica do Rio de Janeiro, Rio de Janeiro, RJ, Brasil.; 10 Renalle Consultoria e Serviços Médicos, Petrópolis, RJ, Brasil.; 11 Universidade Federal do Rio de Janeiro, Rio de Janeiro, RJ, Brasil.; 12 Hospital Santa Casa de Misericórdia de Goiânia, Goiânia, GO, Brasil.; 13 Hospital do Rim, Goiânia, GO, Brasil.; 14 Hospital São Lucas Copacabana, Rio de Janeiro, RJ, Brasil.; 15 Universidade Federal de São Paulo, São Paulo, SP, Brasil.

Prezado Editor,

A publicação do Posicionamento da Sociedade Brasileira de Nefrologia sobre recusa e descontinuação da diálise^1^ supre uma lacuna na literatura nefrológica nacional, oferecendo um referencial técnico e ético para situações em que o tratamento deixa de parecer proporcional ou benéfico ao paciente. O documento confere maior segurança a processos marcados por sofrimento, angústia, incertezas e potenciais conflitos, nos quais se entrelaçam responsabilidade técnica, beneficência, não maleficência, justiça e respeito à dignidade e à autonomia da pessoa com doença renal avançada^2^. 

A diálise não pode ser um imperativo absoluto, mas uma intervenção cuja proporcionalidade deve ser reavaliada à luz da condição clínica, da qualidade de vida e das crenças e valores do paciente. O médico não deve se limitar à dimensão técnica, mas planejar um cuidado que reconheça o paciente como um ser bioético e não como um destinatário de procedimentos.

A reflexão de Santos et al.^3^ destaca acertadamente o caráter autônomo e legítimo das decisões sobre as intervenções, quando tomadas por quem mais importa: o próprio paciente. Ao advertirem que a busca compulsiva por consenso pode obscurecer e até ferir essa prerrogativa, reforçam uma concepção robusta de autonomia, que não se reduz à assinatura de um termo, mas envolve autodeterminação informada, coerente com crenças, valores e projetos de vida. Em consonância com diretrizes internacionais e com uma lei nacional recém-editada^4^, enfatiza-se o direito de recusar ou interromper terapias que não se alinhem aos objetivos do cuidado^5^.

Contudo, a autonomia do paciente pode gerar tensionamento com a corresponsabilidade ética e técnica da equipe. O nefrologista responde pela adequação da indicação e pela avaliação da pertinência ou desproporcionalidade das intervenções. Ao respeitar a decisão do paciente de não iniciar ou descontinuar a diálise, precisa certificar-se de que estão presentes capacidade decisória, esclarecimento e liberdade de escolha e, ainda, que haverá suporte sintomático e paliativo adequado. 

A recusa esclarecida é indiscutivelmente respeitável, ao passo que a recusa baseada em desinformação, desconhecimento, desespero ou coerção sutil exige intervenção (bio)ética do profissional e da equipe. Assim, há que se garantir que o processo decisório seja compartilhado, com comunicação honesta, escuta ativa e documentação transparente das preferências do paciente. A precaução clínica é desejável para que não se configure imperícia, imprudência ou negligência, caso não haja oferta consistente de alternativas de cuidado. A equipe deve respeitar a recusa legítima, bem como considerar que a uremia compromete a autonomia ao contribuir para a vulnerabilidade emocional e cognitiva do paciente, nem sempre evidente.

Finalizando, análises com olhares diversos são bem-vindas e contribuem para qualificar processos decisórios particularmente difíceis, nos quais a equipe, o paciente e seus familiares precisam ponderar proporcionalidade terapêutica, sofrimento, prognóstico, valores pessoais e objetivos de cuidado. Desse modo, se, por um lado, o Posicionamento da SBN valoriza a autonomia do paciente e propõe o consenso como ideal procedimental, por outro, oferece um arcabouço que protege a corresponsabilidade da equipe, permitindo reconhecer, com prudência e coragem moral, o momento em que interromper a diálise possa significar, de fato, cuidar melhor.
